# 61892 Systemic TLR3-targeting Combinatorial Chemokine Modulation Sensitizes Murine Tumors to PD-1 Blockade

**DOI:** 10.1017/cts.2021.444

**Published:** 2021-03-30

**Authors:** Kathleen M. Kokolus, Nataša Obermajer, Per Basse, Pawel Kalinski

**Affiliations:** 1 Roswell Park Comprehensive Cancer Center; 2 UPMC Hillman Cancer Center

## Abstract

ABSTRACT IMPACT: This work will lead to improved efficacy of immunotherapy directly impacting the survival of patients with hard to treat cancers. OBJECTIVES/GOALS: Immune checkpoint inhibitors (ICI) are most effective against ‘hot’ tumors highly infiltrated with cytotoxic T lymphocytes (CTLs) but have not worked well in poorly infiltrated ‘cold’ tumors. Thus, we are working to achieve a pretreatment regimen that will create a favorable immune profile allowing more effective ?PD-1 therapy. METHODS/STUDY POPULATION: BALB/c or C57BL/6 mice were inoculated with CRC murine cells CT26 or MC38, respectively. Mice were inoculated by two injection types: subcutaneous (SC), for systemic therapy, or intraperitoneal (IP), for local therapy. Tumor-bearing mice were given a two dose course of CKM consisting of IFN-? and rintatolimod via IP injection. Following CKM administration, mice were treated with three doses of ?PD-1 via IP injection. Mice were monitored for the kinetics of tumor growth and survival following treatment. The tumor microenvironment of treated mice was analyzed for production of chemokines, inflammatory cytokines and immune cell infiltration. RESULTS/ANTICIPATED RESULTS: CKM consisting of combination IFN-? and rintatolimod, but neither monotherapy alone, sensitized murine CRC tumors to subsequent ?PD-1 treatment. In both CT26 and MC38 tumor-bearing mice, tumor growth was hindered by CKM plus ?PD-1 treatment, independently on the route of treatment (local or systemic). Mice which experienced complete tumor regression were protected from re-challenge with a dose of tumor cells double that of the initial inoculation. Sensitizing tumors to ?PD-1 did not require intratumoral CKM administration and was observed with systemic application at distant sites. In accordance with these observations we expect that systemic CKM will induce strong increases of total and tumor-specific CTL counts in the tumor tissues as measured by both PCR and flow cytometry. DISCUSSION/SIGNIFICANCE OF FINDINGS: CKM sensitizing cold tumors to ?PD-1 indicates that intratumoral CTLs are an important factor dictating therapeutic effectiveness, independent of other factors such as tumor mutational load. The benefit of the sequential short-term CKM followed by routine ?PD-1 make this strategy feasible for rapid inclusion of into routine immunotherapy plans.

